# Impacts of the synthetic androgen Trenbolone on gonad differentiation and development – comparisons between three deeply diverged anuran families

**DOI:** 10.1038/s41598-019-45985-4

**Published:** 2019-07-03

**Authors:** Beata Rozenblut-Kościsty, Maria Ogielska, Juliane Hahn, Denise Kleemann, Ronja Kossakowski, Stephanie Tamschick, Viola Schöning, Angela Krüger, Ilka Lutz, Petros Lymberakis, Werner Kloas, Matthias Stöck

**Affiliations:** 1 Department of Evolutionary Biology and Conservation of Vertebrates, Wroclaw University, Sienkiewicza 21, 50-335 Wroclaw, Poland; 2 Leibniz-Institute of Freshwater Ecology and Inland Fisheries (IGB), Müggelseedamm 301 & 310, D-12587 Berlin, Germany; 3 Natural History Museum of Crete, University of Crete, Knossou Ave., 71409 Heraklion, Crete Greece; 4 Department of Endocrinology, Institute of Biology, Faculty of Life Sciences, Humboldt University, Unter den Linden 6, 10099 Berlin, Germany

**Keywords:** Non-model organisms, Non-model organisms, Non-model organisms, Non-model organisms, Agroecology

## Abstract

Using a recently developed approach for testing endocrine disruptive chemicals (EDCs) in amphibians, comprising synchronized tadpole exposure plus genetic and histological sexing of metamorphs in a flow-through-system, we tested the effects of 17β-Trenbolone (Tb), a widely used growth promoter in cattle farming, in three deeply diverged anuran families: the amphibian model species *Xenopus laevis* (Pipidae) and the non-models *Bufo*(*tes*) *viridis* (Bufonidae) and *Hyla arborea* (Hylidae). Trenbolone was applied in three environmentally and/or physiologically relevant concentrations (0.027 µg/L (10^−10^ M), 0.27 µg/L (10^−9^ M), 2.7 µg/L (10^−8^ M)). In none of the species, Tb caused sex reversals or masculinization of gonads but had negative species-specific impacts on gonad morphology and differentiation after the completion of metamorphosis, independently of genetic sex. In *H*. *arborea* and *B*. *viridis*, mounting Tb-concentration correlated positively with anatomical abnormalities at 27 µg/L (10^−9^ M) and 2.7 µg/L (10^−8^ M), occurring in *X*. *laevis* only at the highest Tb concentration. Despite anatomical aberrations, histologically all gonadal tissues differentiated seemingly normally when examined at the histological level but at various rates. Tb-concentration caused various species-specific mortalities (low in *Xenopus*, uncertain in *Bufo*). Our data suggest that deep phylogenetic divergence modifies EDC-vulnerability, as previously demonstrated for Bisphenol A (BPA) and Ethinylestradiol (EE2).

## Introduction

Among the complex reasons for global amphibian decline (like industrial agriculture, habitat destruction, invasive species, climate change, land use, and infectious diseases), endocrine disruptive compounds (EDCs) are suspected to play a role in the multiple stress syndrome that this vertebrate class experiences^1–6^. EDCs comprise “natural products or synthetic chemicals that mimic, enhance or inhibit the action of hormones, and thus interfere with the synthesis, secretion, transport, binding, action, or elimination of natural hormones, which are responsible for the maintenance of homeostasis, reproduction, development, and/or behavior”^7^.

While many EDCs rather present byproducts of medical or industrial processes, some steroids are widely used as growth promoting substances in cattle farming in the Americas, Australia, and China. The most commonly applied synthetic androgen presents Trenbolone (hereafter: Tb), which has been used to increase muscle mass from the seventies of the twentieth century^8–10^. Nowadays, it is supplied by injection or implant in form of Tb acetate^11–14^, which has an 8–10 times stronger anabolic activity and a 2–5 times stronger androgenic activity than testosterone and is metabolized to biologically active 17α- and 17β-Tb^15^. When tested in human MDA-kb2 cell cultures, the isoform 17α was about 20 times less androgenically active compared to the isoform 17β^16^ but had almost the same activity on the fathead minnow *Pimephales promelas*^17^.

Both isoforms are excreted with cattle feces in concentrations from 5 to 75 ng/g, and their half-life was measured to be about 260 days^12,18^. In tanks collecting manure on farms, the concentration of Tb achieved 1000 ng/L^19^, and in surface waters around farms the concentration of 17α-Tb was found to be 20–50 ng/L while that of 17β-Tb reached 4–6 ng/L^20^.

As shown in several previous studies, released Tb-metabolites can have masculinizing effects also for water-dependent aquatic and semi-aquatic vertebrates. Embryos and larvae of the channel catfish *Ictalurus punctatus*^21^, blue tilapia *Oreochromis aureus*^20^, zebrafish *Danio rerio*^10,22^, black crappie *Pomoxis nigromaculatus*^23^, and guppies *Poecilia reticulata*^24^, developing in Tb-containing waters, differentiated to all-male monocultures. Other examples are female fathead minnows *Pimephales promelas*^13^ and Japanese medaka *Oryzias latipes*^25^ that developed external male features when exposed to Tb.

Amphibians are at high risk to be exposed to various environmental endocrine disruptors, especially during their larval development and/or their post-metamorphic life in aquatic habitats. Typically, under the influence of sex hormones, synthesized by somatic tissues according to genetic sex, their early bipotential gonads differentiate into either testes or ovaries^26^. The susceptibility of amphibians to endocrine disruptors has been well documented, resulting in sex reversal, intersexes, mixed sexes, gonadal developmental abnormalities and/or sterility. Among others, this has been shown for feminizing substances, such as 17α or β-ethinylestradiol and BPA. Examples come from *Xenopus laevis*^27–32^, *Bombina bombina* and *Bombina variegata*^29^, *Hyla arborea*^29–32^, *Bufo(tes) viridis*^29–32^, *Lithobates sylvaticus*^33^, *Euphlyctis cyanophlyctis*^34^, and *Rana curtipes*^35^. On the other hand, vulnerability to masculinizing substances has been tested using testosterone, for example in *Rana rugosa*^36^, *Rana japonica*^37^, *Pelophylax nigromaculatus*^34^, and *Rana curtipes*^35^, and often resulted in sex reversals. In *X*. *laevis*, 10^−8^ M testosterone did not affect sex ratio, whereas methyltestosterone and dihydrotestosterone at 10^−8^ M shifted it to a higher proportion of males^38^. Adverse impacts of (anti-)estrogenic and (anti-)androgenic EDCs, including the androgen methyldi–hydrotestosterone, administered at 10^−8^ M, have been assessed in adult *X*. *laevis* of both sexes, demonstrating impacts on gonad histomorphology^39^, sex steroid levels and EDC biomarkers^40^ as well as on gene expression of gonadotropins and gonadotropin releasing hormone^41^. After its detection at amphibian breeding sites, few tests have included 17β-Tb as an environmentally relevant endocrine disruptor^42,43^. In *X*. *laevis*, reduced bodies mass and size were described^43^. Furthermore, in *X*. *tropicalis*^44^, and *Pelophylax nigromaculatus*^14^, high tadpole mortality, higher male-to-female-biased ratio, and intersexes were observed. In addition, male nuptial pads developed in both sexes of *X*. *laevis* after completion of metamorphosis, i.e. eight weeks earlier than usual^44^.

Some of us have recently developed a novel methodology for EDC testing in amphibians, comprising synchronized tadpole exposure of representatives of three systematic anuran families and pioneering genetic and histological sexing of metamorphs in a flow-through-system^30^, with which we have, so far, tested two feminizing substances: EE2 and BPA^30–32^. This approach allowed for the first time the immediate comparison of EDC effects on the sexual development of three deeply divergent model and non-model anurans under identical experimental conditions.

Therefore, the aim of the present study was two-fold: first, we wanted to apply this synchronized exposure and genetic sexing approach to a potentially masculinizing agent. Secondly, we focused on the effects of 17β-Tb on mortality of tadpoles and the degree (stage) of gonadal differentiation after completion of metamorphosis in three phylogenetically deeply diverged taxa: an amphibian model species, the African clawed frog (*Xenopus laevis*), and two non-model species, the European green toad (*Bufo viridis*) and the European tree frog (*Hyla arborea*). As the rate of gonadal development in each of these species differs^45^, we expected various impacts of this potentially masculinizing, environmental endocrine disruptor on the differentiating ovaries and testes.

## Results

### Stability of experimental design

**Figure 1:**
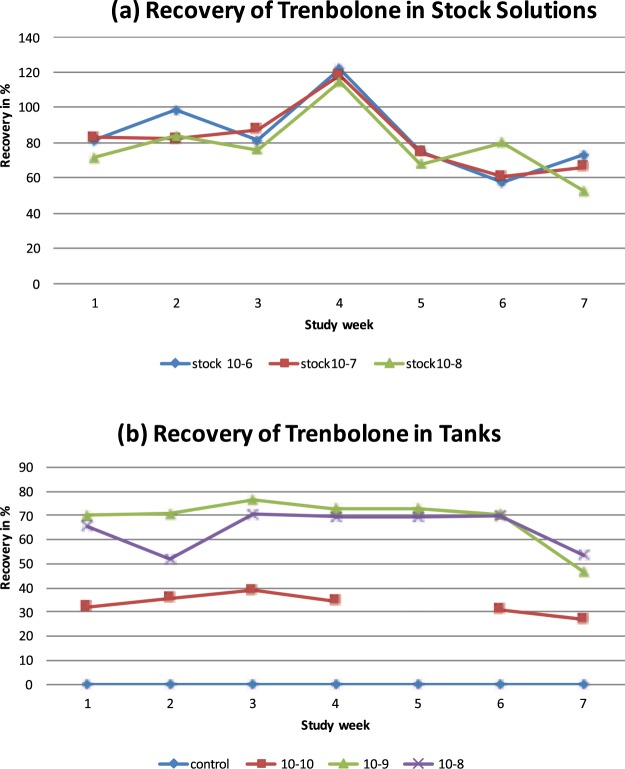
Monitoring of concentrations of trenbolone. **(a)** Recovery of Trenbolone in stock solutions; **(b)** recovery of Trenbolone in tanks. Note that in (**b**) in week 5, due to a technical issue during samples processing, data for 10^−10^ M are missing. Nominal Tb-concentrations in tanks (**b**) comprised (0.027 µg/L (10^−10^ M, red), 0.27 µg/L (10^−9^ M, green), 2.7 µg/L (10^−8^ M, violet). Controls were free of Tb, apart from one measurement in study week (0.05 ng/L in one tank); see text for technical details on measurements.

Monitoring of Tb concentration levels in the tanks revealed some deviations from the nominal concentration, reaching from 27% to 39% for 10^−10^ M, from 46% to 76% for 10^−9^ M, and from 53% to 70% for 10^−8^ M (Fig. 1). Other water parameters (cf^46^.) were more stable and are shown in Table 1.

**Table 1:** Monitoring of water parameters in experimental tanks.

	Desired values	Measured values	Mean
Temperature [°C]	22 ± 1	21.0–23.8	21.7
Dissolved O_2_ [%]	≥60	72.4–99.9	95.36
pH	7.9–8.3	6.9–8.2	7.9
Light intensity [lx]	100–500	213–660	344
NH_4_ [mg/l]	0.00–0.35	<0,05–0,15	0.06
NO_3_ [mg/l]	0.04–2.29	<0,5–2,25	1.66
hardness	9–13	10–13	11.03

### Mortality

**Figure 2:**
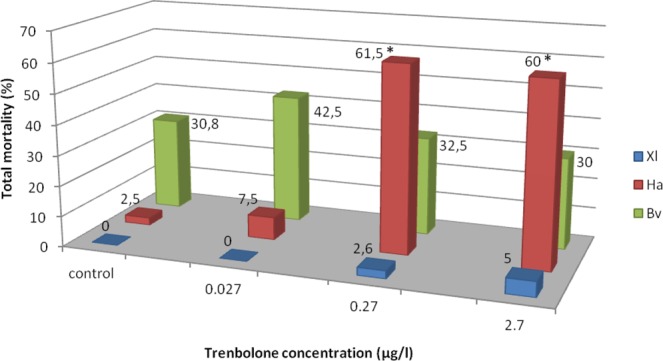
Mortality of tadpoles and metamorphs under three Trenbolone concentrations in percent. Xl: *Xenopus laevis*, Ha: *Hyla arborea*, Bv: *Bufo viridis*, *significant difference between control and treatment group within the same species (2-sided Chi^2^-tests, p < 0.05).

Mortality was assessed from tadpole to post-metamorphic development until the end of the experiment and exhibited species-specificity. The lethal rate of *Xenopus laevis* tadpoles at concentrations of 10^−9^ M and 10^−8^ M was 2.6% and 5%, respectively, but the differences between the control and Tb-treated groups were not significant [10^−10^ M (p = 0.33), 10^−9^ M (p = 0.31), and 10^−8^ M (p = 0.15)]. *Hyla arborea* tadpoles’ mortality rate in the control series comprised 2.5%, reached 7.5% at the lowest Tb-concentration of 10^−10^ M, and increased at higher Tb-concentration, comprising 61.5% at 10^−9^ M and 60% at 10^−8^ M. Mortality was equal between control and Tb 10^−10^ M (p = 0.31) but significant differences occurred between controls and Tb 10^−9^ M and 10^−8^ M (p < 0.005). In *B*. *viridis*, dead individuals were recorded at all Tb-concentrations and among the control. Total mortality for this species ranged from 30% (10^−8^ M) to 42.5% (10^−10^ M) (Fig. 2, Table 2). The difference was species-specific and statistically significant (Chi^2^-test for species pairs, df = 1, p < 0.05). However, in one series of *B*. *viridis* (replicate 1), we observed high mortality, probably caused by an infection or genetic effect, both in the control and in all experimental replicates. However, differences between the control and experimental series were not significant, both when replicate 1 was included [10^−10^ M (p = 0.28), 10^−9^ M (p = 0.87), and 10^−8^ M (p = 0.940)] and when replicate 1 was excluded [10^−10^ M (p = 0.64), 10^−9^ M (p = 0.87), and 10^−8^ M or (p = 0.47)].

**Table 2:** Summary of effects on gonadal development of African clawed frogs (*Xenopus laevis*), European tree frogs (*Hyla arborea*) and European green toads (*Bufo viridis*) in control groups and treatments 0.027 µg/L (Tb10^−10^ M), 0.27 µg/L (Tb 10^−9^ M), and 2.7 µg/L (Tb 10^−8^ M) of Trenbolone.

*Xenopus laevis*	Control		0,027 μg/l (10^−10^ M)		0,27 μg/l (10^−9^ M)		2,7 μg/l (10^−8^ M)	
	N	%	N	%	N	%	N	%
Total mortality	0	0	0	0	1	2.6	2	5.0
Mortality of tadpoles	0	0	0	0	1	2.6	2	5.0
Mortality of metamorphs	0	0	0	0	0	0	0	0
Genetic females	23	57.5	16	39.0	19	50.0	20	50.0
Genetic males	17	42.5	25	61.0	19	50.0	20	50.0
Impaired gonadal gross morphology	0	0	0	0	0	0	5^a^	12.5
Shortened gonad (anatomy)	0	0	0	0	0	0	3	7.5
Discontinuous gonad (anatomy)	0	0	0	0	0	0	2 (1)*	5.0
Sterile gonads (histology)	2	5.0	0	0	2	5.3	2	5.0
***Hyla arborea***								
Total mortality	1	2.5	3	7.5	24^a^	61.5	24^a^	60.0
Mortality of tadpoles	1	2.5	3	7.5	8	20.5	8	20.0
Mortality of metamorphs	0	0	0	0	16	41.0	16	40.0
Genetic females	17	42.0	20	54.9	6	40.0	10	62.5
Genetic males	23	57.5	17	45.1	9	60.0	6	37.5
Impaired gonadal gross morphology	0	0	1	2.7	3^a^	20.0	4^a^	25.0
Shortened gonad (anatomy)	0	0	1	2.7	2	13.3	4	25.0
Discontinuous gonad (anatomy)	0	0	0	0	1*	6.7	0	0
Sterile gonads (histology)	0	0	0	0	1	6.7	0	0
***Bufo viridis***								
Total mortality	12	30.8	17	42,5	13	32.5	12	30.0
Mortality of tadpoles	0	0.0	2	5.0	4	10.0	2	5.0
Mortality of metamorphs	12	30.8	15	37.5	9	22.5	10	25.0
Genetic females	14	51.9	8	36.4	12	44.4	12	42.9
Genetic males	13	48.1	14	63.6	15	55.6	16	57.1
Impaired gonadal gross morphology	6	22.2	10	45.5	16^a^	59.3	15^a^	53.6
Shortened gonad (anatomy)	6 (3)*	22.2	7 (3)*	58.3	9 (3)*	33.3	9 (2)*^,#^	32.14
Discontinuous gonad (anatomy)	0	0	1*	4.5	5 (3)*^,a^	18.5	8 (5)*^,#,a^	28.6
Doubled Bidder’s organ/organ or part of Bidder’s on the testes (anatomy)	0	0	2	9.1	2	7.4	10^#^	35.7
Partly sterile gonads (histology)	0	0	0	0	0	0	1	4,2
Degenerated Bidder’s organ (histology)	0	0	3	15.8	0	0	2	8.4

()*Only one gonad affected, ^#^fragmented testis and doubled Bidder’s organ, or short end gonad and doubled Bidder’s organ were visible together. ^a^Significant difference between control and treatment group within the same species (2-sided Chi^2^-tests, p < 0.05).

### Phenotypic sex, gonadal anatomy and histology

In all experimental animals, phenotypic sex was consistent with genotypic sex, irrespective of Tb concentrations. No intersexes and sex-reversals were recorded, however, in 3 genetic *X*. *laevis* females (10^−8^) and 2 female *B*. *viridis* (10^−8^), initially, gonads were anatomically considered as testes, but histologically clearly identified as ovaries. In these 5 individuals, however, ovaries were short and ovarian lobes were not well developed. The Tb influence on gonad gross anatomy differed between species. In *X*. *laevis*, 12.5% were “impaired” (for definition: see Materials and Methods) at the highest Tb (10^−8^ M) and the difference was statistically significant in comparison to the control (Chi^2^-test, p = 0.03). The two non-model species did not differ from controls at 10^−10^ (*H*. *arborea* p = 0.31; *B*. *viridis* p = 0.06), but differed from treatments at two higher concentrations [*H*. *arborea* 10^−9^ M (p = 0.036), 10^−8^ M (p = 0.026); *B*. *viridis* 10^−9^ M (p = 0.001), and 10^−8^ M (p = 0.013)].

**Figure 3:**
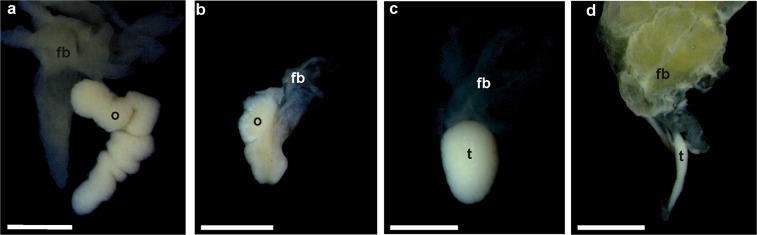
Photographs of anatomically normal and small gonads of *Hyla arborea*. (**a**) Normal ovary (control), (**b**) shortened ovary (10^−8^), (**c**) normal testis (control), (**d**) small testis (10^−9^). Scale bars represent 1 mm, fb – fat body, o – ovary, t – testis.

**Figure 4:**
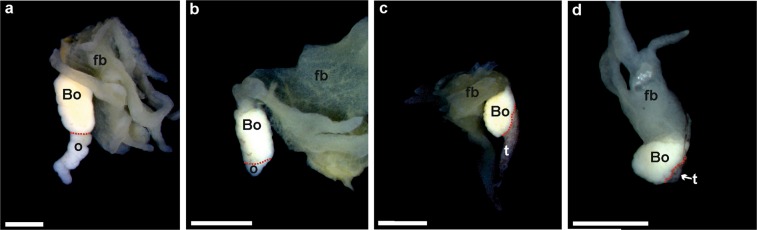
Photographs of anatomically normal and small gonads of *Bufo viridis*. (**a**) Normal ovary (control), (**b**) shortened ovary (10^−8^ M), (**c**) normal testis (control), (**d**) small testis (10^−9^ M). Scale bars represent 1 mm; the red dotted line marks the boundary between Bidder's organ and the gonad. Bo – Bidder’s organ (occurring only in bufonid gonads), fb – fat body, o – ovary, t – testis.

Gonads with reduced sizes becoming more as 60% shorter than control groups, were considered as shortened due to the influence of Tb. Ovaries with species-specific numbers of thick lobes, as judged from controls, were considered as normally developed; fewer or shorter lobes were classified as shortened. This kind of shortened gonads, properly assessed by anatomy and histology, were observed in the experimental series and their frequency increased along with the Tb concentration (Table 2). In *H*. *arborea*, shortened testes were observed in one male at concentration 10^−10^ M, two males at 10^−9^ M, and four males at 10^−8^ M (Fig. 3). In *B*. *viridis*, shortened gonads were observed in four males and three females at 10^−10^ M, four males and five females at 10^−9^ M, and three males and six females at 10^−8^ M (Fig. 4). Shortened gonads were also observed in the control series of *B*. *viridis* in three males and three females.

**Figure 5:**
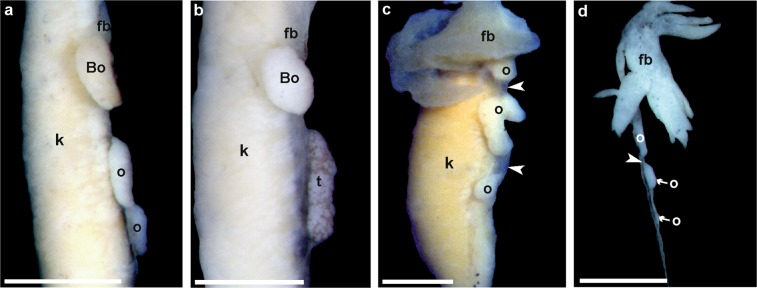
Photographs of fragmented (discontinuous) (**a**) ovary (10^−8^) and (**b**) testis (10^−9^) of *Bufo viridis*, (**c**) ovary in *Hyla arborea* (10^−9^) and (**d**) ovary in *Xenopus laevis* (10^−8^). Scale bars represent 1 mm. Note mesovarium indicated by white arrow head, Bo – Bidder’s organ (occurring only in bufonid gonads), fb – fat body, k – kidney, o – all parts of the ovary are marked, t – testis.

**Figure 6:**
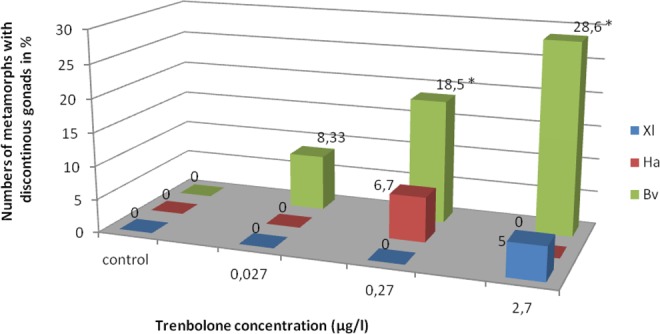
Frequency of discontinuous gonads of *Xenopus laevis* (Xl), *Hyla arborea* (Ha), and *Bufo viridis* (Bv) after treatment with three concentrations of Trenbolone. *Significant difference between control and treatment group within the same species (2-sided Chi^2^-tests, p < 0.05).

Discontinuous gonads consisted of two distinct portions separated by “empty” mesenthery (mesovarium or mesorchium; Fig. 5). This anatomical abnormality did not affect proper differentiation as assessed by histology. The feature occurred in two female *X*. *laevis* (at Tb 10^−8^ M) and one female *H*. *arborea* (at Tb 10^−9^ M). Both sexes were affected in *B*. *viridis*: one male at 10^−10^, two males and three females at 10^−9^, and two males and six females at 10^−8^; in 64% only one (mainly the left) gonad was discontinuous. The frequency of these gonads is presented in Fig. 6.

**Figure 7:**
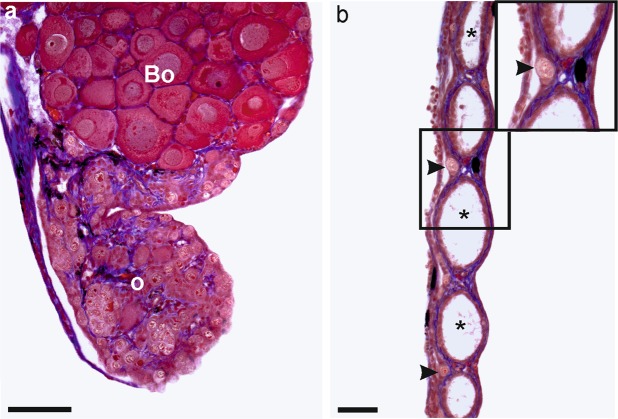
Histological sections of sterile ovary in (**a**) *Bufo viridis* (10^−8^) and (**b**) *Xenopus laevis* (10^−9^). Visible intense degeneration of germ cells in *B*. *viridis* ovary – o. Scale bars represent 100 µm. Bo – Bidder’s organ (characteristic of bufonid gonads), *ovarian cavity, arrow heads – single oogonia in ovary cortex.

**Figure 8:**
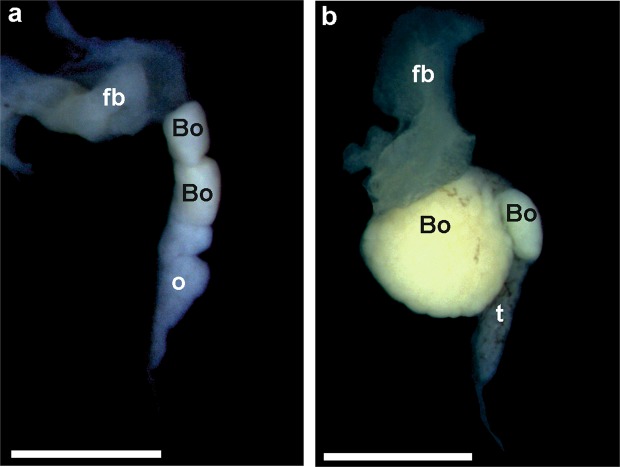
Anatomical photographs of doubled Bidder’s organ (Bo) in (**a**) ovary (10^−10^) and (**b**) testis (10^−10^) of *Bufo viridis*. Scale bars represent 1 mm, fb – fat body, o – ovary, t – testis.

**Figure 9:**
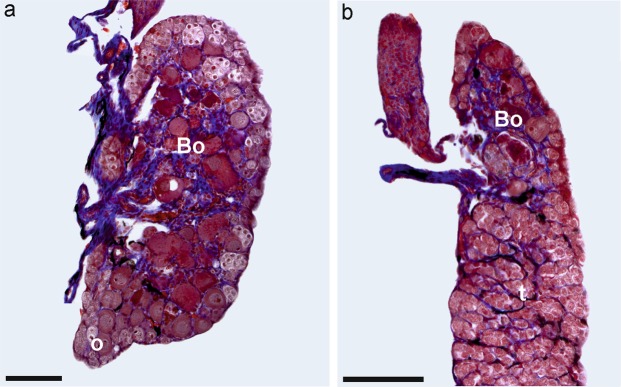
Histological sections of degenerated Bidder’s organ (Bo) in (**a**) ovary (10^−10^) and (**b**) testis (10^−10^) of *Bufo viridis*. Scale bars represent 100 µm, o – ovary, t – testis.

Bidder’s organ (BO) is an anterior ovary-like portion of the gonad, which differentiates very early during testis and ovary development in bufonids. In *B*. *viridis*, BO is observed by gross morphology and histology (Fig. 7a). In most green toads, BOs were normal, but in two males at Tb 10^−10^ M, two males at Tb 10^−9^ M, and 10 males at 10^−8^ M, BOs consisted of two parts, a larger and smaller one (Fig. 8, Table 2). In two males and one female at 10^−10^ M and one male and one female at 10^−8^ M, BOs were small and contained small previtellogenic oocytes (Fig. 9).

Impaired gonads in *Hyla* and *Xenopus* were either shortened or discontinuous gonads. In *Bufo,* the impaired gonads occurred either alone (8 males had doubled Bidder’s organ, 2 males and 3 females had short gonads, 1 male and 4 females had discontinuous gonads) or combined with the double Bidder’s organ (1 male had shortened gonads and double Bidder’s organ, and 1 male had discontinuous gonads and double Bidder’s organ). In 2 females, the ovaries were shortened and discontinuous.

Sterile gonads with none or considerably reduced numbers of germ cells were rare and were observed in two female *X*. *laevis* at 10^−9^ M and two females at 10^−8^ M, two female *H*. *arborea* at 10^−9^ M, and one *B*. *viridis* at 10^−8^ M (Fig. 7). Sterile gonads were also observed in one male and one female in a control series in *X*. *laevis*.

The degree of gonad development and differentiation (i.e., gonadal stage) was species-specific and was less advanced in *X*. *laevis* and *B*. *viridis* compared to *H*. *arborea* (Table 3). In both non-model species, the differentiation stage differed between control and Tb-treated gonads. In *X*. *laevis*, in the control and animals exposed to Tb 10^−10^ M and 10^−9^ M, most ovaries (87.5–91.7%) and testes (62.5–100%) were at stage V, some ovaries (8.3–12.5%) at stage IV, and 4 testes at stage VI. In Tb 10^–8^ M, 70% of testes were less advanced and only reached stage IV. Ovaries of *B*. *viridis* were at stage V (55.6–66.6%) or VI (33.3–44.4%), irrespective of the Tb concentration. The higher the Tb concentration, the more advanced testes were observed. At Tb 10^−9^ and 10^−8^, 50% testes were at stage VII, and 50% at stage VIII. Less advanced testes were observed in the control: stage VI (36.4%), VII (36.4%), and VIII (27.2%). Ovaries of *H*. *arborea* were at stages VIII (50–81.8%) or IX (18.2–50%), regardless of the Tb-concentration. Testes in control and Tb 10^−10^ were at stage IX (100%) but were less advanced at higher Tb concentrations (in 10^−9^, 50% at stage VII, 25% at stage VIII, and 25% at stage IX, and in 10^−8^, 16.7% at stage VI, 50% at stage VII, 33.3% at stage VIII) (Table 3). Differences in the advancement of gonadal stage were noticed only in *H*. *arborea* between control and Tb 10^−10^, and the highest Tb concentration (control/Tb 10^−8^ p = 0.006, Tb 10^−10^/Tb 10^−8^ p = 0.011); in the other cases, these differences were not significant.

**Table 3:** Stages of ovaries and testes development of African clawed frogs (*Xenopus laevis*), European tree frogs (*Hyla arborea*) and European green toads (*Bufo viridis*) in control groups and treatments 0.027 µg/L (Tb10^−10^ M), 0.27 µg/L (Tb 10^−9^ M), and 2.7 µg/L (Tb 10^−8^ M) of Trenbolone.

Stage of gonadal development*	Control		0,027 μg/l (10^−10^ M)		0,27 μg/l (10^−9^ M)		2,7 μg/l (10^−8^ M)	
*Xenopus laevis*	N (%) ovaries	N (%) testes	N (%) ovaries	N (%) testes	N (%) ovaries	N (%) testes	N (%) ovaries	N (%) testes
Stage IV	1 (11.1)	2 (25.0)	1 (12.5)		1 (8.3)	1 (9.1)	2 (25.0)	7 (70.0)
Stage V	8 (88.9)	5 (62.5)	7 (87.5)	10 (100)	11 (91.7)	7 (63.6)	6 (75.0)	3 (30.0)
Stage VI		1 (12.5)				3 (27.3)		
***Hyla arborea***								
Stage VI								1 (16.7)^**a**, **b**^
Stage VII						4 (50.0)		3 (50.0)^**a**, **b**^
Stage VIII	6 (60.0)		9 (81.8)		3 (60.0)	2 (25.0)	5 (50.0)	2 (33.3)^**a**,**b**^
Stage IX	4 (40.0)	9 (100)	2 (18.2)	7 (100)	2 (40.0)	2 (25.0)	5 (50.0)	
***Bufo viridis***								
Stage IV	1 (8.3)							
Stage V	7 (58.3)		4 (57.1)		5 (55.6)		7 (58.3)	
Stage VI	4 (33.4)	4 (36.35)	3 (42,9)		4 (44.4)		5 (41.7)	
Stage VII		4 (36.35)		8 (72.7)		6 (50.0)		6 (50.0)
Stage VIII		3 (27.2)		3 (27.3)		6 (50.0)		6 (50.0)

^*^According to Ogielska and Kotusz (2004) and Haczkiewicz and Ogielska (2013). ^a^Significant difference in gonadal development between control and treatment group Tb 10^−8^ and ^b^significant difference between treatment groups Tb 10^−10^ and Tb 10^−8^ in *Hyla arborea* (Kruskal-Wallis-H-Tests, p < 0.05).

## Discussion

Since 1989 (modified in 2003), the European Union has prohibited the production or import of meat and meat products from animals treated with estradiol-17β, testosterone, progesterone, zeranol, trenbolone acetate or melengestrol acetate. Nevertheless, discussions about unification of agricultural standards were ongoing during the recently dropped TTIP negotiations (https://en.wikipedia.org/wiki/Transatlantic_Trade_and_Investment_Partnership), and namely in the World Trade Organization (WTO), topics are now mostly focusing on effects on human health. However, steroids, when released or leaking into ecosystems, can have many side effects on aquatic species and – as we show here – would have a number of detrimental effects in European amphibians as well. In this respect, our study might be a warning sign to prevent the use of this substance, regularly applied on four continents, in the future agriculture of the European animal production.

**Figure 10:**
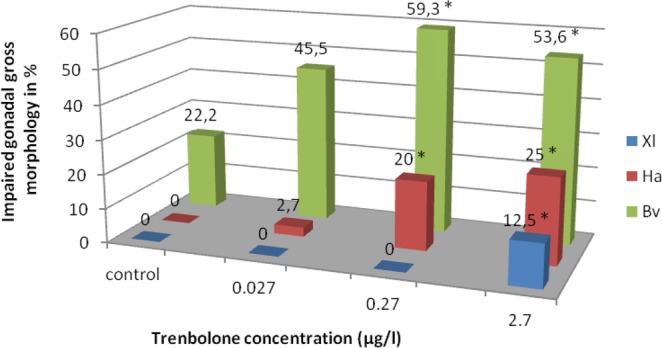
Frequency of impaired gonads of *Xenopus laevis* (Xl), *Hyla arborea* (Ha), and *Bufo viridis* (Bv) after treatment in various concentrations of Trenbolone. *Significant difference between control and treatment group within the same species (2-sided Chi^2^-tests, p < 0.05).

Here, we show a number of gonadal abnormalities and variable tadpole mortality in three amphibian species (a model species *X*. *laevis* and two non-models *H*. *arborea* and *B*. *viridis*), subjected to Tb-treatment. Effects comprised a dosage-dependent impact on *Hyla*, a strongly significant effect in *Bufo* and only a moderate effect in *Xenopus* (only at the highest Tb concentration of 10^−8^ M), which demonstrated strong species-specificity (Fig. 10). However, despite the observed anatomical defects, all gonads differentiated seemingly normally, but in various rates, depending on species and Tb-concentration.

### Stability of experimental design and limitations

We used the same high standard flow-through system as in our former experiments involving EE2^30,32^ and BPA^31^. During the Tb-exposure, we experienced some fluctuations in the maintenance of the nominal Tb-concentration. Similar observations (at concentrations of 0.005, 0.05, and 0.5 µg/L) were reported by Ankley *et al*.^13^, most probably due to deposition of Tb inside the pipe system; the other suggested reason could be that Tb is differently metabolized by tadpoles^46^. The lowest recorded Tb-concentration in our experimental system was lower than in the environment of drains on cattle farms, i.e. 20 ng/L (0.02 µg/L)^42^ or in experiments using *X*. *tropicalis* (0.078–0.1 µg/L)^44^. Therefore, our results demonstrate the physiological relevance of Tb-concentrations, detected in the environment. Other water parameters were maintained constantly, especially pH, which may cause developmental abnormalities and mortality, as shown for pH < 5.5 in *Hyla cinerea*^47^ and *Rana temporaria*^48^. Meeting the challenge of synchronically exposing model and non-model amphibians included collecting similarly developed eggs in the field (*Hyla*) or finding naturally ready-to-reproduce adults, then reproducing under controlled conditions (*Bufo*), transferring them to the lab (from Greece to Germany) and raising them for weeks along with same-aged larvae of *Xenopus*. However, co-exposure in 24 tanks, as a potential merit of our study, came at the expense of having relatively few replicates (two per exposure regime or control group and per species), making our statistical results less robust.

### Mortality

Olmstead *et al*.^44^ revealed almost 100% mortality of tadpoles of *X*. *tropicalis* at 0.31 and 1.25 µg/L 17β-Tb; even at the lowest concentration (0.1 µg/L), the mortality was high (40%). Li *et al*.^14^ also stated high mortality for 0.1–10 µg/L 17β-Tb in *Pelophylax nigromaculatus* tadpoles. Another androgen, testosterone propionate, at a concentration of 30 µg/L had the same effect on *X*. *laevis* tadpoles^49^. In all cases, the tadpoles died during during and after completion of metamorphosis and displayed hypertrophy of larynx muscles and disorders in the respiratory and cardiac systems. The larynx of *Xenopus* is sexually dimorphic, allowing the emission of mating calls by males; androgen-treatment accelerates the larynx development, which in turn blocks air flow into the lungs and directs it to the intestine^44^. Trenbolone-treated *P*. *nigromaculatus* tadpoles exhibited abnormal swimming and improper metamorphosis, probably due to anomalous muscle development^14^.

In contrast to the aforementioned experiments, we did not observe high mortality in *X*. *laevis*, similar to Haselman *et al*.^50^. We found only 5% *X*. *laevis* tadpole mortality during and after completion of metamorphosis, even under Tb-concentrations twice as high (2.7 μg/L) as in *X*. *tropicalis*, where mortality reached 100%^44^. Among the two non-model species, we observed significantly higher mortalities during and after metamorphic climax. In *H*. *arborea*, mortality reached ca. 60% under higher Tb-concentrations (0.27 and 2.7 μg/L). Despite (naturally except for the treatment) identical rearing conditions like in our previous experiments involving EE2 and BPA^30–32^, in the present trials we observed increased mortality in green toads. However, this mortality in *B*. *viridis* (ca. 30%) remains ambiguous, because we recorded similar results in one of the control series, presumably caused by an infection or a genetic effect. Unfortunately, the high mortality in the *B*. *viridis* control series prevented us from showing clear correlation between gonadal development and Tb toxicity in this species. High mortality was correlated with higher incidence of gonadal developmental retardation and abnormalities. In summary, we can say that five amphibian species studied so far exhibit various mortalities under Tb-treatments, and thus again marked species-specific effects.

### Phenotypic sex, gonad anatomy and histology

Undifferentiated amphibian gonads are bipotential and differentiate into ovaries or testes due to the action of sex hormones^26,45,51^. Environmental xenohormones can imitate and/or modify sex hormones (resulting in intersexes, sex reversals, and sterility) and/or impair the gonad differentiation. In *X*. *laevis*, *B*. *viridis*, and *H*. *arborea*, we have previously observed such effects of the feminizing agents Bisphenol A or 17 α-Ethinylestradiol^30–32^. In contrast, the potentially masculinizing Tb neither led to sex reversal nor masculinization of ovaries; in all treated (and control) individuals the genetic and phenotypic sexes were consistent. Sterility was only recorded in females (5.1% *X*. *laevis*, 6.7% *H*. *arborea*, and 4.2%. *B*. *viridis*) and thus might be consistent with the action of a masculinizing endocrine disruptor, however it was rare and therefore we cannot claim that it was an effect of Tb. Our data are similar to experiments in *X*. *laevis*^50^. Nevertheless, treatment with higher concentrations of Tb (0.078–10 µg/L) led to masculinization, sex-ambiguous gonads, and intersexes as reported for *P*. *nigromaculatus*^14^ and *X*. *tropicalis*^44^.

We paid special attention to the histology of gonads because pure anatomical observations may lead to incorrect sexing biased to males, especially when gonads are reduced in size (shortened). We found substantial differences among the studied species in response to various Tb-dosages. *Xenopuslaevis* was the most resistant (12.5% individuals displayed gonadal impairments in the highest Tb-concentration), whereas the two non-model species, *H*. *arborea* and *B*. *viridis*, were sensitive to all Tb-concentrations (25% *H*. *arborea* and 53.6% *B*. *viridis* at the highest concentrations). The most common anomaly was a reduced size of otherwise normally differentiated gonads, but with fewer seminiferous tubules in testes or thinner cortex in ovaries; this in turn may cause reduced fertility in adults. Reduced size of gonads (mainly testes) was also observed in *B*. *viridis* and *H*. *arborea* in our previous experiment examining BPA^31^.

In all species, we observed a peculiar abnormal discontinuity of gonads along the rostral–caudal axis, similar to that described by Carr *et al*.^52^ and Hayes *et al*.^53^ in *X*. *laevis* and Coady *et al*.^54^ in *Rana clamitans*, after atrazine treatment (0.1–25 µg/L). The fragments of gonads were not connected to each other and were separated by mesovarium or mesorchium. This abnormality was most common in *B*. *viridis* (28.6%) of both sexes but affected only females in *H*. *arborea* and *X*. *laevis*. Hayes *et al*.^53^ proposed the name “polygonadism” for this abnormality, however, we would rather avoid this name because it suggests multiple gonads, while there are only two but discontinuous ones. We suppose that the possible cause of this malformation was a result of disturbed migration of primordial germ cells (PGCs) into the gonadal ridges during the early stages of gonadal development, which roughly coincides with the very beginning of the Tb-treatment. In the absence of PGCs, the somatic portion of a gonad fails to differentiate or degenerates^55^ and thus gives rise to separated gonadal fragments.

Independently from any kind of malformations in the three species studied, Tb did not affect the stage of ovary differentiation, as it did in testes. The testes exhibited retarded stages of gonad development in *X*. *laevis* and *H*. *arborea*, as also seen for testosterone treatments in *Bombina bombina*, *B*. *variegata*, *X*. *laevis*, *H*. *arborea*, and *B*. *viridis*^29^. However, we observed an accelerated rate of testes development in *B*. *viridis*. We also analyzed Bidder’s organ (BO), an ovarian-like structure in the proximal portion of the bufonid gonads, sensitive to ovarian and testicular hormones^56^. Testosterone-treatment caused an inhibition of the BO-differentiation during metamorphic climax and even its atrophy in *Bufo japonicus* (*vulgaris*) *formosus*^57^, *Duttaphrynus* (*Bufo*) *melanostictus*^58^ and in *B*. *viridis*^29^. However, after Tb-treatment, we observed that the majority (92.6%) of *B*. *viridis* had normal BO, and only in three males and two females the oocytes inside BO degenerated.

### Amphibians *vs*. fish as indicators for endocrine disruptive chemicals (EDCs)

The masculinizing effect of Tb has so far mainly been studied in fish and concerned modifications of phenotypic features and gonad anatomy^10,13,21–24,59,60^. Seki *et al*.^25^ described masculinization of secondary sex characteristics in female Japanese medaka (*O*. *latipes*) at a Tb-concentration of 0.365 µg/L and in fathead minnow at a Tb-concentration 0.401 µg/L. Trenbolone also affects mating behaviour, namely female choice; female guppies (*Poecilia reticulata*), treated 21 days with 0.004 ng/L of Tb, spent much less time among males than untreated ones^61^.

Adult teleost fish, in contrast to amphibians, are more sensitive to the masculinizing effects of Tb on gonads and thereby may serve as a good indicator for environmental pollution by EDCs. At least some fish species can naturally change gender (sex) and display sequential hermaphroditism and sex reversal more than once during their ontogenesis (reviewed by Hamlett^62^). This may be caused by differences in gametogenesis, especially oogenesis. Females of the major extant vertebrate clades represent two basic modes of oocyte recruitment. In amphibians, similar to mammals, proliferation of primary oogonia (gonocytes) is limited and all oocytes are formed during early ontogenesis, once for the entire life span and are recruited from a finite stock of resting follicles at consecutive breeding seasons^63^; in this case the source of new oocytes is “closed”. In some teleosts, however, proliferation of gonocytes is unlimited (cyclic) and the oocyte stock is *de novo* created after each spawning; thus, the source of new oocytes remains “open” during the entire life span^64,65^. This was confirmed for the medaka *Oryzias latipes*^66^, the very long-lived scorpaenid genus *Sebastes*, which produce new generations of oocytes even when 70–80 years old^67^, the syngnathids *Syngnathus scovelli* and *Hippocampus erectus*^68^, and cichlid *Tilapia aurea*^69^. On the other hand, a “closed ovary” was reported for the poecilid guppy, *Poecilia reticulata*^70,71^.

The “closed” type of ovary is correlated with gonochorism, *i*.*e*. permanent and non-reversible determination of separate male or female sex during early ontogenesis, as also typical of anuran amphibians (for review^72^). The “open ovary” seems to have offered new opportunities in evolutionary flexibility of reproduction due to the unlimited supply of new generations of undifferentiated gonocytes during the whole reproductive life span of an individual.

To the best of our knowledge, information about increased mortality of fry and adult fishes caused by Tb are lacking^10,13,20–25,59,60^.

In summary, we suggest that some species of bony fishes (*Oryzias latipes*, *Tilapia aurea*) may serve as good bioindicators to test EDCs on gonads in adults, whereas in amphibians this process is restricted to tadpole and juvenile stages. On the other hand, mortality of larvae seems easier to be studied in anuran amphibians due to a clearly marked metamorphosis.

## Conclusions

Due to unexpected (and from previous experiments under identical conditions unknown) high mortality affecting *B*. *viridis* (including the control groups), the synchronized exposure to the potentially masculinizing Tb agent was not entirely successful across all three species. This was especially unfortunate as in this species, the effect of Tb on gonadal development showed the strongest effect and appears not to be correlated with overt toxicity, since all *B*. *viridis* groups exhibited mortality similar to the control, but gonadal differentiation issues were higher in exposure groups in a dose-dependent fashion. Generally, irrespective of the concentration, Tb caused neither sex reversal nor masculinization of gonads in any of the three studied amphibian species but had adverse species-specific impact on gonadal development. Especially at higher concentrations, the two non-model species, *H*. *arborea* and *B*. *viridis*, were more Tb-prone than the model *X*. *laevis*. This suggests that deep phylogenetic divergence modifies endocrine disruptive vulnerability as also shown by Tamschick *et al*.^30–32^. Based on our data, we conclude that one cannot easily predict the sensitivity to EDCs studying model species only. As shown in this and other studies on EDCs, mortality and almost all somatic (but not gonadal) defects in all amphibian species occurred during and after completion of metamorphosis. Therefore, we cannot rule out that xenohormones also influence the thyroid gland that is the source of T3 and T4, the hormones that play a pivotal role in the control of somatic but not gonadal development during metamorphosis (for reviews^73,74^).

## Materials and Methods

### Animals

*Xenopus laevis* tadpoles were obtained from the stock at the Leibniz-Institute of Freshwater Ecology and Inland Fisheries (IGB). Induction of spawning and tadpole husbandry followed standard methods^75^. Parental animals of *B*. *viridis* and *H*. *arborea* were caught at several localities in Greece (Table 4), and non-invasively DNA-sampled^76^. Parts of their clutches were transferred to IGB (permit 115790/229) and acclimated at 22 ∓ 1 °C in 10 L Milli-Q grade water, supplemented with 2.5 g marine salt (Tagis, Germany). Siblings of different genetic families of *B*. *viridis* and *H*. *arborea* were assigned to different exposure or control replicates.

**Table 4:** Origin, Sample IDs, replicate numbers, and numbers of experimental animals.

Species	Locality (site of origin)	Geographic Coordinates	Sample ID	Replicate number	Number of individuals
*Hyla arborea*	Greece, Crete, Thrapsano	35.178 25.28	Ha_Thrapsano1	1	40 siblings and 40 mixed offspring
		35.175 25.304	Ha_Thrapsano2	2	80 siblings
*Bufo viridis*	Greece, Crete, Lasithi Plateau	35.187N 25.196E	Bv_GR15_1♀ × 2♂	1	80 siblings
			Bv_GR15_3♀ × 4♂	2	80 siblings
*Xenopus laevis*	Lab stock, IGB		Xl_Gr. 8	1	80 siblings
			Xl_Gr. 9	2	80 siblings

European tree frogs (*Hyla arborea*) and European green toads (*Bufo viridis*) were obtained from wild populations; African clawed frogs (*Xenopus laevis*) came from a lab stock.

### Hormone exposure and experimental conditions

17β-Trenbolone (17β-Hydroxyestra-4,9,11-trien-3-one, Sigma-Aldrich, Germany) was dissolved in dimethyl sulfoxide (DMSO 99.5%; Roth, Germany) and applied in concentrations of 0.027 µg/L (Tb 10^−10^ M), 0.27 µg/L (Tb 10^−9^ M), and 2.7 µg/L (Tb 10^−8^ M). Control animals received 0.00001% DMSO. Tb concentrations in test tanks were checked weekly by high performance liquid chromatography/mass spectrometry (UHPLC-QTOF) and adjusted if required. Analyses were performed on an Agilent 1290 infinity / 6550 iFunnel with a Dual AJS ESI ion source in positive mode within a mass range from 100 to 1500 m/z. Chromatographic conditions were as follows - column: Agilent Eclipse Plus C18 (2.1*50 mm 1.8 µm) at 30 °C, flow: 400 µL/min; solvent A: 0,1% formic acid, solvent B: 10 mM ammonium acetate in methanol; gradient: 0 min 20% B, 10 min 80% B, 11 min 20% B, post run 2.5 min. Due to the Tb detection limit of 5 ng/mL water samples had to be concentrated by solid phase extraction (SPE). The Thermo Fisher Scientific SolEx C18, 0.5 g cartridges were automatically processed using a Dionex Autotrace 280 (Thermo Scientific), conditioned with 6 mL methanol and 6 mL water; water samples, prior filtered through glass fiber filters, were transferred simultaneously through the cartridges, followed by a drying step with nitrogen for 20 min and eluted with 3 mL methanol and 4 mL dichloromethane. Samples were evaporated at 40 °C to dryness under a gentle stream of nitrogen using a rotary evaporator. Residues were re-dissolved in methanol. To minimize adsorption or release of EDCs, we used glass tanks and all connections of the flow through system consisted of inert materials involving mainly PTFE (Polytetrafluoroethylene, “Teflon”)-coating or Platinum-cured silicon tubing (Cole-Parmer). In *B*. *viridis* and *H*. *arborea*, exposure of tadpoles started when they began to swim independently, i.e. at Gosner^77^ stages 22–23 (equivalent to Nieuwkoop and Faber^78^ stages 42–44 in for *X*. *laevis*), which is distinctly prior to the sensitive phase of sex determination in all species^51,79^. Twenty randomly chosen individuals per species and treatment were transferred into each test tank in a high-standard flow-through-system (for details^46^). Two replicates per exposure group (including control) comprised in total 160 tadpoles per species (Table 4). Stock solutions and water were piped via a peristaltic pump into a mixing chamber, mixed to final concentrations, and supplied to a cluster of three test tanks each. Concentrations were thus identical for all three species in each treatment group. Tadpoles were reared in a 12/12 h light/dark cycle at constantly 22 ± 1 °C in sufficiently aerated and regularly cleaned tanks. Weekly monitored water parameters comprised: dissolved oxygen, nitrate, ammonium, pH, conductivity, and hardness; values were adequate as in previous studies, involving the same equipment. Tadpoles were fed SeraMicron (Sera, Germany), *H*. *arborea* and *B*. *viridis* were additionally supplied with TetraMin (Tetra, Germany). To imitate natural conditions, under which *H*. *arborea* and *B*. *viridis* leave water at metamorphosis, animals were transferred to glass terraria at Gosner stage 46 (metamorphosis completed). *Xenopus laevis* were dissected at equivalent Nieuwkoop-Faber stage 66, while *H*. *aborea* and *B*. *viridis* were examined after sufficient post-metamorphic differentiation^45^. For these two species, terraria contained bowls with pure water that were cleaned two to three times a week. Postmetamorphic animals were fed with *Drosophila*.

### DNA extraction, PCRs and genotyping

DNA extractions were performed using a Qiagen BioSprint 96 DNA Plant Kit together with the BioSprint robotic workstation (Qiagen, Germany) at the Museum für Naturkunde (Leibniz-Institute for Evolution and Biodiversity Research, Berlin). DNAs were eluted in 200 mL AE buffer (Qiagen) and stored at −20 °C. To establish genetic sex, species-specific polymerase chain reactions (PCRs) were conducted with subsequent gel electrophoresis and genotyping. Genetic sexing of *H*. *arborea* and *X*. *laevis* was conducted as described by Tamschick *et al*.^30^, sexing of *B*. *viridis* involved markers C201, C223, and D214^80^ (Table 5). Only frogs and toads that completed metamorphosis were genetically sexed. Dead tadpoles were excluded from all further analyses (except to quantify for mortality approach) because gonads were not suitable for histology. For detection of potential sex reversals, the genotype according to the sex-linked markers and the phenotype of each metamorph were directly compared.

**Table 5:** Primer information for molecular sexing of *Xenopus laevis*, *Hyla arborea* and *Bufo viridis*.

Species	Locus	GenBank Acc. number	Primer sequences (5′=>3′)	Fluores- cence label	Allele size (bp)
*X*. *laevis*	*DMRT1*	AB259777	F: AACAGGAGCCCAATTCTGAG R: AACTGCTTGACCTCTAATGC	none	206
*X*. *laevis*	*DM-W*	AB365520 F:	F: CCACACCCAGCTCATGTAAAG R: GGGCAGAGTCACATATACTG	none	260
*B*. *viridis*	C201	HQ386137	F: AGGACCCAGGATTTCCAT R: GCTTCTACCAAAGACTGTTCC	HEX (green)	94–122
*B*. *viridis*	C223	FJ613135	F: CAGAGGTCAAGAGGGAGAAG R: GGCACCACATCCTGATTAG	FAM (blue)	164–200
*B*. *viridis*	D214	JX658773	F: CACTGTGCAGGCGCAACT R: CGTATGCTGTTTTCTTCTGTG	ATTO (black)	252–276
*H*. *arborea*	Ha-H108	EU029102	F: GGGGGTGAGTAAGGGTTAAATC R: GCCACTGTATAGTCCCTCCCTA	FAM (blue)	234–261
*H*. *arborea*	WHa5–201	AJ403999	F: TCATGGACTGTCGTCATGGT R: AGGTAAATGGAATCTGGGTGTG	HEX (green)	228–253

Markers with corresponding accession numbers, sequences, fluorescence labels and sizes in base pairs (bp) for all three species. Note that markers for *B*. *viridis* and *H*. *arborea* can only be applied for sexing if the parental sex and genotypes are known.

### Dissection, anatomical and histological examination

Animals were euthanized by immersion in tricaine methanesulfonate (MS222; Sigma-Aldrich) and then killed by decapitation. Thigh muscle tissue was stored in 100% ethanol at −20 °C for later DNA extraction. After opening the body cavity, gonads were examined using a stereo microscope (Olympus SZX7) with an attached Olympus DT5 camera. Based on gross gonadal anatomy, phenotypic sexes were identified and the presence or absence of morphological abnormalities recorded (Table 2). After examination at the anatomical level, gonads were referred to as “impaired” if their shape deviated from that of normally developing ovaries and testes typical of each species, and as found in controls (shortened, discontinuous and, in the case of *Bufo*, with doubled Bidder’s organ). Other deviations, such as increased degeneration of germ cells, sterility or partial sterility, were described only at the histological level and were not classified as “impaired gonads”. Gonad length was determined on the basis of calibrated pictures, taken twice: first at dissection (*in situ*) when we had the opportunity to examine the mutual location of kidneys, gonads and fat bodies and second, when cleaned from adjoining tissues before embedding in paraplast. Knowing normal shape and size of gonads at a given stage (metamorphosis completed) for a given species, we were able to classify them as “shortened” or normal. We estimated the length of gonads by the use of scale bars, added automatically to each picture. Because gonads naturally vary in size between individuals, we classified gonads as “shortened”, when they constituted 40% or less of the “normal” length of gonads (i.e. observed in normally developing froglets known to us from wild populations (for details^45^). To prepare gonads for histology, they were fixed in natural anatomical positions, together with adjacent body parts, in Bouin’s solution (Sigma) for 24 h, rinsed several rounds in 70% EtOH until the solution was no longer yellowish, and finally stored in EtOH (70%) until further processing. The gonads were then separated from adjoining tissues and photographed using Stemi SV11 (Zeiss) microscope and a cooled Carl Zeiss Axio-Cam HRc CCD camera. Anatomically visible size deviations of gonads between control and exposed animals were recorded using scaled macro-photographs.

Histological sections were prepared for 50% of all study animals (10 randomly chosen individuals per tank, i.e. 20 per experimental group) without prior information on genetic sex.

For histology sections, gonads were embedded in Paraplast using standard procedures, sectioned on Leica RM 2255 microtome into 7 μm-thick longitudinal sections, stained with Mallory’s trichrome, and examined using a Zeiss Axioskop 20 microscope. Images were acquired by a cooled Carl Zeiss Axio-Cam HRc CCD camera. Stages of gonadal development were assessed according to Haczkiewicz and Ogielska^45,51,81^. Females were characterized by typical ovarian structures (oogonia, early oocytes, diplotenes, ovarian cavities), while male gonads were recognized by testicular structures (spermatogonia, spermatocytes, seminiferous tubules, rete testis). Histological samples were screened slide by slide for detection of rate of differentiation, possible alterations in morphology or sex reversal.

### Statistical analyses

Statistical analyses were performed with STATISTICA 13.5 software (StatSoft, Poland). In order to compare control and exposure groups within and between species (mortality, gonadal gross morphology), we used cross-tabulations with 2-sided Chi square tests (α = 0.05). Initially, for each parameter replicates of controls or treatments were compared. If no significant differences (exact p = 0.05) were found, we pooled both replicates and compared control and exposure groups. Kruskal-Wallis-H-tests and multiple (double-sided) comparisons were used for stages of gonadal development/treatment comparisons.

### Ethics statement

This experiment was approved by the German State Office of Health and Social Affairs (LaGeSo, Berlin, Germany; G0359/12); all treatments were carried out in accordance with approved guidelines at the IGB, according to the LaGeSo-permit as well as the animal welfare committee’s instructions.

## Data Availability

All data are included in the publication.
